# Excessive Ostial Stent Protrusion: Evaluation of Management Strategies and Clinical Outcomes of the Side Flap Technique

**DOI:** 10.1002/ccd.70545

**Published:** 2026-03-10

**Authors:** Gregor Leibundgut, M. Bilal Iqbal, Claudiu Ungureanu, Anastasios Barmpas, Jasper Boeddinghaus, Teodor Buliga, Gabor G. Toth, Alexandru Achim

**Affiliations:** ^1^ University Heart Center, University Hospital Basel Basel Switzerland; ^2^ Department of Cardiology Royal Jubilee Hospital Victoria British Columbia Canada; ^3^ Department of Cardiology Hôpital de Jolimont, YG La Louvrièr La Louvière Belgium; ^4^ Interbalkan Medical Centre Thessaloniki Greece; ^5^ Cardiology Department, Heart Institute University of Medicine and Pharmacy “Iuliu Hatieganu” Cluj‐Napoca Romania; ^6^ University Heart Center Graz Medical University Graz Graz Austria

**Keywords:** aorto‐ostial lesions, drug‐eluting stents, percutaneous coronary intervention, Side Flap technique, stent protrusion

## Abstract

**Backgrounds:**

Accurate stent positioning in aorto‐ostial lesions remains challenging due to complex three‐dimensional anatomy, lack of optimal fluoroscopic projections, and high rates of geographic miss. Stent overhang impairs guide catheter re‐engagement and complicates future revascularization. Despite its frequency, no standardized approach exists for managing excessive stent protrusions.

**Aims:**

This study systematically evaluated management strategies for excessive aorto‐ostial stent protrusion, integrating comprehensive bench testing with clinical case experience, and assessed the feasibility, mechanics, and outcomes of the Side Flap technique.

**Methods:**

Bench experiments assessed multiple corrective strategies in two scenarios, guidewire crossing through the central stent lumen and through a side cell, across contemporary DES platforms. Mechanical feasibility, force transmission, stent deformation and expansion, and structural integrity were evaluated using high‐pressure ballooning, catheter‐assisted maneuvers, and intracoronary imaging. A retrospective multicenter series provided complementary real‐world clinical outcomes of the Side Flap technique.

**Results:**

Longitudinal stent compression using guide catheters, large balloons, or telescoping maneuvers proved mechanically ineffective and frequently produced severe stent deformation or malapposition. In contrast, intentional side‐cell recrossing with creation of a neo‐lumen was consistently feasible, structurally stable, and reproducible. All DES platforms tolerated large‐diameter side‐cell expansion without strut fracture, yielding circular neo‐lumens with excellent apposition on OCT. Clinically, the Side Flap technique achieved high procedural success with no technique‐related adverse events during follow‐up.

**Conclusions:**

The Side Flap technique offers a controlled and reproducible alternative strategy when true‐lumen access is not achievable or fails, and represents a valuable addition to the interventional armamentarium. Longitudinal stent crush techniques are unreliable for managing excessive aorto‐ostial protrusion.

**List of Abbreviations**
CAU: Caudal
CRA: Cranial
CT: Computed Tomography
DES: Drug‐Eluting Stent
F: French (guiding catheter size)
IVUS: Intravascular Ultrasound
JR: Judkins Right (guiding catheter)
LAO: Left Anterior Oblique
LM: Left Main (coronary artery)
MI: Myocardial Infarction
OCT: Optical Coherence Tomography
PCI: Percutaneous Coronary Intervention
POT: Proximal Optimization Technique
RCA: Right Coronary Artery
TAVI: Transcatheter Aortic Valve Implantation
TLF: Target Lesion Failure
WALPO: Wire in the Aorta for Localization and Protection of the Ostium

## 1 Introduction

Accurate stent positioning in aorto‐ostial coronary lesions remains technically demanding and frequently suboptimal, owing to the intrinsic limitations of two‐dimensional angiography and substantial interindividual anatomical variability [1]. Longitudinal geographic miss, resulting from incorrect stent length or positioning, is particularly consequential in aorto‐ostial interventions, with reported misplacement rates between 54% and 87% [2, 3, 4]. The right coronary artery (RCA) is more frequently affected. A CT‐based “double S‐curve” analysis demonstrated marked variability in the perpendicular projection of the RCA ostium compared to the left main (LM), with optimal RCA angles ranging from LAO 49°−79° and CAU 42°−61°, versus LM angles of LAO 33°−40° and CRA 19°−25° [1]. In practice, these optimal projections were achievable in only 8% of RCA cases, compared with 80% for the LM [1].

Patients with longitudinal geographic miss face a two‐ to threefold higher rate of target vessel revascularization (TVR) compared to those achieving precise ostial coverage [3, 5]. Excessive stent length further worsens outcomes by increasing late luminal loss (LLL) and longitudinal stent deformation (LSD) [6, 7, 8]. Moreover, stent protrusion into the aorta impedes coaxial catheter engagement, predisposes to stent crushing during future procedures, and complicates reintervention, particularly when the overhang exceeds 5 mm, which severely limits re‐access and re‐treatment at this site. Despite the frequency and clinical relevance of this problem, no standardized approach exists for managing an overhanging stent. Current clinical strategies include true‐lumen rewiring with ostial balloon optimization, side‐cell techniques, and, in selected cases, stent extraction. However, each approach has inherent limitations and may require additional re‐stenting, thereby increasing the risk of recoil or recurrent protrusion [9, 10, 11].

This study systematically evaluated several bench techniques for managing excessive aorto‐ostial stent protrusion, addressing two typical scenarios: guidewire passage through the central stent lumen and guidewire crossing via a side strut. Complementary real‐world cases are presented to illustrate the feasibility and potential limitations of these approaches.

## 2 Technical Considerations for Optimal Aorto‐Ostial Stent Positioning

Careful attention is required when positioning a stent in aorto‐ostial lesions. Ideally, the stent should protrude no more than 1 mm (approximately one stent ring) into the aorta, as greater protrusion provides no advantage and significantly impairs future catheter engagement and re‐intervention. Conversely, geographic miss with incomplete ostial coverage often necessitates the implantation of a second stent, resulting in multiple metallic layers at the ostium, a recognized driver of target lesion failure (TLF). Furthermore, implantation of a short ostial stent in such scenarios can be technically challenging and carries a considerable risk of misplacement or renewed protrusion. Partial ostial coverage carries a similar risk.

Several technical considerations are critical for achieving optimal results during aorto‐ostial PCI:

(A) Projection planning: Owing to the marked anatomical variability of the coronary ostia, no universal standard projection exists. Coronary CT can help define the optimal ostial projection and, with the expanding use of CT‐based pre‐procedural planning, may offer accurate fluoroscopic angles to facilitate precise stent implantation in routine practice [1].

(B) Aortic flaring effect: “Nailing the ostium” can be misleading, as either proximal optimization technique (POT) or aortic flaring typically elongates rather than shortens the stent by approximately 1–3 mm, depending on balloon size [12]. Consequently, an initial protrusion of 1–2 mm may increase to 2–4 mm after postdilatation. This effect can be mitigated by using short POT balloons and by avoiding distal‐first inflation, instead performing proximal POT first [13].

(C) Ineffective bidirectional flaring: Contrary to common belief, repeated balloon inflations in upward and downward directions do not meaningfully flare the stent. Due to the stent's plasticity, it simply follows the balloon shaft and remains in its position after balloon deflation and equipment removal.

(D) Patient stability: Controlled breath‐holding remains the most fundamental and effective method to reduce diaphragmatic motion and optimize stent positioning accuracy.

(E) Implantation technique: The stent should always be deployed in a pull‐back configuration, never in push mode. The guiding catheter should remain freely floating outside the ostium and sufficiently disengaged from the stent balloon to minimize the risk of uncontrolled “melon‐seed” displacement of the catheter or stent during balloon inflation. Contrast injections should be performed from this position to prevent hydraulic coronary or aortic dissection.

(F) WALPO technique: A simple and effective method to reliably delineate the true aorto‐coronary ostium using a secondary guidewire [14]. In addition to accurately identifying the ostial location, WALPO enhances guiding catheter stability and minimizes the risk of deep intubation. The auxiliary wire should be advanced far enough to ensure that the soft, radiopaque tip segment does not prolapse into the coronary lumen.

(G) The floating balloon technique: This variation of the WALPO enhances stability by inflating a 2.0–2.5 mm coronary balloon over the aortic guidewire and pressing it gently against the aortic wall [15, 16]. This configuration improves coaxiality but introduces catheter crowding. Caution is required when retracting the deflated aortic balloon to avoid dislodging the newly deployed stent.

(H) IVUS‐assisted positioning: Intravascular ultrasound (IVUS) can provide superior ostial visualization and facilitate precise stent placement under real‐time guidance [17, 18]. Although cardiac motion, IVUS catheter displacement, and friction‐related coupling between the IVUS catheter and the stent can complicate the procedure, several technical refinements can substantially mitigate these challenges. Positioning the floating wire downward within the aortic cusp improves stability, while the use of larger‐lumen 7 Fr guiding catheters reduces friction and facilitates smoother device manipulation. Despite its technical complexity, IVUS‐guided ostial stenting represents a superior strategy for precise stent positioning and should be preferentially adopted rather than dismissed based on a steeper learning curve.

(I) Dedicated devices: Ostial PRO Stent Positioning System (Merit Medical Systems, South Jordan, UT, USA) disposable, guidewire‐based alignment device featuring expandable radiopaque nitinol or gold‐plated feet that brace against the aortic wall to mark the ostial plane [19]. Once the stent delivery system crosses the lesion, the device is advanced until the feet expand (“power position”), physically blocking deeper catheter advancement and defining the aorto‐coronary junction. Accurate front‐ or back‐loading and careful fluoroscopic guidance are essential.

(J) Minimizing stent movement: Breath holding may help, but “pistoning” is often amplified in bradycardia or with short stents, increasing the risk of misplacement. Temporarily raising the heart rate pharmacologically or via transcoronary pacing can mitigate this effect [20].

## 3 Strategies for Re‐Engagement in Aorto‐Ostial Stent Protrusion

Excessive aorto‐ostial stent protrusion is commonly defined as extension > 5 mm into the aortic lumen or presence ≥ 3 protruding stent rings, corresponding to an overall length of approximately 3.6–4.5 mm, based on a typical ring spacing of 1.2–1.5 mm in contemporary DES platforms [21, 22]. Beyond this threshold, coaxial catheter engagement becomes exceedingly difficult. This limitation stems from the intrinsic behavior of the guiding catheter, which tends to drift toward the aortic wall during rotation. Consequently, the catheter preferentially aligns either above or below the protruding stent rather than precisely along its central axis, a challenge further compounded by the three‐dimensional orientation of the stent axis and the limited fluoroscopic visibility of its true lumen.

In this context, inadvertent wiring through a side cell may initially go unrecognized. Suspicion typically arises only when the catheter appears non‐coaxial or when resistance is encountered during advancement of coronary devices.

Although wiring through a side cell is often faster and technically simpler, this approach effectively creates a neo‐lumen while deflecting the overhanging stent segment toward the aortic sinus. This “flapped” configuration has been hypothesized to increase the risk of adverse events, such as stent or aortic thrombosis and embolic stroke, although definitive clinical evidence remains lacking. The subsequent sections present additional data and bench observations addressing this concern.

Conversely, when the aorto‐ostial stent protrusion is moderate (approximately 1–5 mm), the operator may attempt to re‐enter the true stent lumen using a series of controlled maneuvers:

(A) Use of small, non‐selective guiding catheters: A non‐selective guiding catheter, typically a Judkins Right type with a smaller diameter (5–6 Fr) and a curve of 3.5–4.0, should be used. The reduced size improves maneuverability within the aortic root and minimizes the risk of inadvertent deep engagement or deformation of the protruded stent (floating guide catheter).

(B) Gentle and controlled movements: Manipulation of the guiding catheter should rely primarily on rotation rather than on push‐pull movements. Rotational adjustments minimize the risk of abrupt stent contact or dislodgement of the protruded segment. An anti‐clockwise rotation, for example, withdraws an AL 0.75 guiding catheter from the RCA ostium.

(C) Anchoring technique using dual guidewires: One guidewire is first inserted through a side strut of the protruding stent to anchor and stabilize the guiding catheter. A second guidewire is then advanced at a distance from the stent, ideally with a loop or knuckle configuration, to seek entry into the true stent lumen. The second wire should advance with less resistance than the first, although fluoroscopic separation between both wires may be subtle due to the three‐dimensional anatomy.

(D) Dual‐lumen microcatheter‐facilitated wiring: This recently described method represents an evolution of the wire‐based anchoring technique [23]. A dual‐lumen microcatheter is advanced over the initial “false” side‐strut guidewire. Through the secondary lumen, a second guidewire is directed under improved control toward an alternative path, ideally traversing the true stent lumen. The use of angulated microcatheters may further enhance steerability and facilitate selective wiring in challenging anatomies. However, when first engaging a protruding aorto‐ostial stent with the intention of achieving true‐lumen rewiring, the dual‐lumen microcatheter should be advanced over the aortic cusp wire rather than over an initial false side‐strut wire [24]. In this configuration, the initial wire is parked in the aortic cusp, serving as a stable rail for the monorail segment of the dual‐lumen catheter, while a second wire is subsequently advanced through the over‐the‐wire lumen to selectively access the true stent lumen. This strategy provides superior catheter stability, minimizes inadvertent engagement of side struts, and increases the likelihood of successful true‐lumen wiring at the first attempt [24].

(E) Intracoronary imaging confirmation: Before performing further maneuvers, the exact guidewire position should be verified with intracoronary imaging to confirm re‐entry into the true stent lumen and avoid subsequent complications.

(F) Role of pre‐procedural CT: Pre‐procedural coronary CT imaging remains valuable in this context, as it allows precise quantification of the stent's aortic protrusion and aids in selecting the most suitable strategy for re‐entry.

## 4 Management of Ostial Stent Protrusion

Management of aorto‐ostial stent protrusion depends on both the extent of stent overhang and the site where the guidewire crosses the stent. Whenever feasible, true‐lumen rewiring should be prioritized, followed by restoration of stent geometry using an ostial‐sized non‐compliant balloon. This approach is particularly suitable in cases of moderate protrusion and stent endothelialization, whereas longer protrusions are more likely to be crossed through a side cell and therefore require alternative management strategies.

## 5 Guidewire Crossing Through the Central Stent Lumen

If the guidewire is positioned within the central stent lumen, one theoretical approach involves performing a longitudinal stent crush to achieve mechanical foreshortening, bringing adjacent stent rings closer together to close inter‐ring gaps or even partially telescope the stent segments into one another. While conceptually attractive, the critical question is whether a truly controlled and effective longitudinal crush can be achieved in practice without inducing significant stent deformation, malapposition, or adding excessive metal burden within an ostium that is already compromised.

### 5.1 Ostial Flaring

Ostial flaring using a large balloon is frequently performed to ensure adequate stent apposition at the typically tapering coronary ostium. However, due to the inherent stent elongation that occurs under high‐pressure overexpansion [12], this maneuver may paradoxically worsen ostial protrusion rather than correct it.

The Flash™ Ostial System (Ostial Corporation, Sunnyvale, CA, USA) is a dedicated flaring device composed of a high‐pressure, coronary‐sized balloon and a larger, low‐pressure anchoring balloon that can expand up to 14 mm [19]. It is intended to flare the stent struts protruding 1–2 mm beyond the ostium, thereby improving future catheter engagement during angiography and re‐intervention. However, given the large diameter of its secondary balloon and the limited overexpansion capacity of coronary stents relative to this size, the protruding stent rings may not truly flare but instead become longitudinally compressed or crushed toward the ostium.

### 5.2 Intentional Longitudinal Stent Deformation

At first glance, longitudinal compression of the protruding stent struts may appear to offer a potential solution for managing excessive aorto‐ostial protrusion. Over the years, numerous techniques have been attempted in clinical practice to achieve this effect, mostly anecdotal and lacking systematic validation.

#### 5.2.1 Direct Guide Catheter Crushing

**FIGURE 1:**
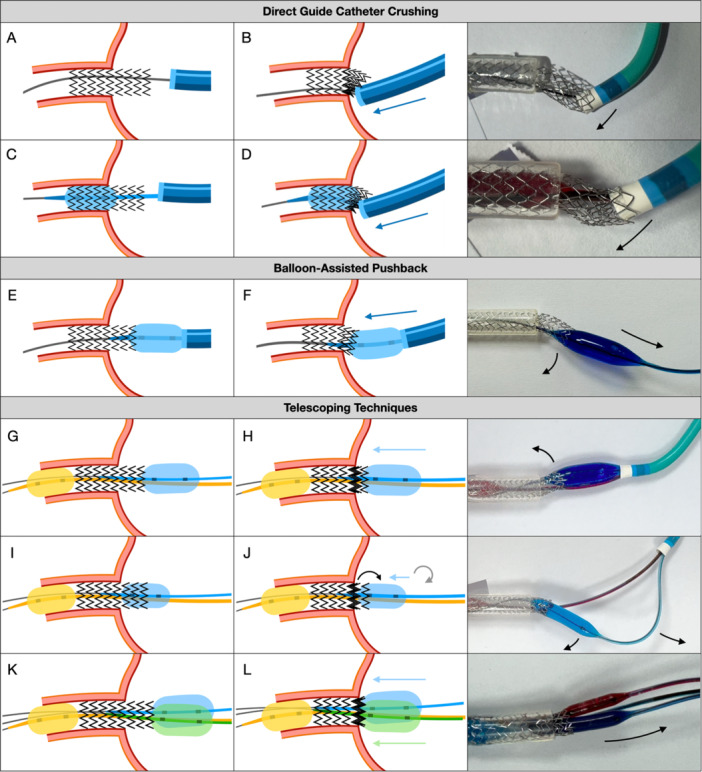
Illustrations of Longitudinal Crushing Techniques. (A–B): Direct guide catheter crushing: Engaging and pushing the guiding catheter against the protruding stent to reduce overhang. (C–D): Anchor‐assisted guide push: Inflation of a distal anchoring balloon to enhance guide support and pushability. (E–F): Balloon‐assisted pushback: Inflation of a large balloon within or outside the protruded stent and forward pushing. (G–H): Telescoping technique: An anchoring balloon within the stent pulls another one outside the protruding segment toward it, generating an “active crush” more aligned with the stent's axis. (I–J): Sequential telescoping (“ring‐by‐ring”) technique. (K–L): Dual‐balloon telescoping: Two parallel smaller balloons positioned outside the protruding stent segment are simultaneously pulled toward it. [Color figure can be viewed at wileyonlinelibrary.com]

Engaging the protruding stent with the guiding catheter and pushing it toward the ostium may seem like a simple corrective maneuver to reduce protrusion (Figure 1A,B). To enhance guide pushability, an anchor balloon can be inflated distally within the stent (Figure 1C,D). However, bench testing in both scenarios demonstrated that this approach is mechanically unfeasible. The applied force is not transmitted along the stent's longitudinal axis but directed downward toward the aortic valve. Increasing push force causes immediate guide catheter disengagement, while the balloon induces eccentric stent deformation. Instead of achieving repositioning, the maneuver flips the stent downward, further compromising the ostium and distorting the ostial struts, resulting in persistent deformation and malapposition. Although additional ostial balloon inflations may occasionally correct malapposition, advancement of a new balloon can be hindered by the distorted stent architecture, and re‐dilatation often leads to renewed stent elongation.

#### 5.2.2 Balloon‐Assisted Pushback

Inflating a balloon within the protruding portion of the stent and pushing it longitudinally toward the ostium may appear to be a logical corrective maneuver. However, when a large balloon is advanced in this manner, the applied force is again not transmitted along the stent's longitudinal axis but rather directed downward toward the aortic valve. As the pushing force increases, the guiding catheter rapidly disengages, while the balloon causes lateral deformation of the stent without generating any controlled forward movement along the intended axis. (Figure 1E,F).

#### 5.2.3 Telescoping Technique

In this approach, an anchoring balloon is used to pull the balloon positioned within the protruding stent segment toward it. This maneuver generates an active crush effect, directing the applied force more favorably along the stent's longitudinal axis and aiming to minimize the gap between the two balloons. Despite the improved force alignment, the crush remains somewhat uneven, with part of the energy still dissipating laterally. After forceful pulling and pushing, the larger balloon frequently causes partial invagination of the stent, beyond which additional compression becomes ineffective. The effectiveness of this technique may be further limited by partial entrapment of the anchoring balloon within the protruding stent struts by the opposing pushing balloon. Nonetheless, the overall result is superior to that achieved with the simple pushback technique (Figure 1G,H).

In cases of very long stent protrusion, the technique was performed sequentially in a “ring‐by‐ring” manner (Figure 1I,J). In this setting, maintaining coaxial alignment is technically impossible. After initial partial success, the telescoping balloon typically causes disengagement of the guiding catheter, leading to outward deflection of the balloon shaft. Subsequent repositioning of the balloon more proximally reproduces the limitations of other pushing techniques, as any further inflation from this point fails to achieve true longitudinal stent compression. The applied forces cannot be precisely controlled, preventing the creation of a uniform telescoping effect.

**FIGURE 2:**
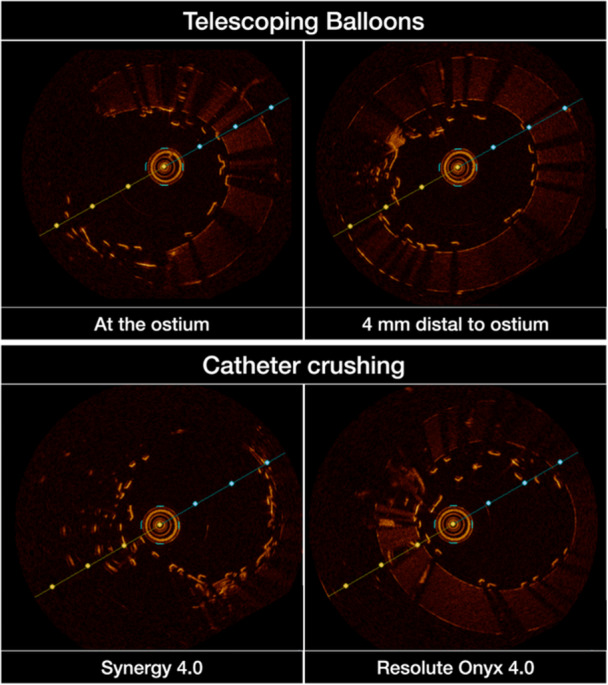
Assessment of ostial stent apposition after longitudinal crushing techniques. Optical coherence tomography (OCT) images demonstrate longitudinal stent deformation (strut crowding) at the coronary ostium with clear evidence of malapposition. [Color figure can be viewed at wileyonlinelibrary.com]

A modified telescoping technique using two parallel balloons within the protruding stent segment was also evaluated (Figure 1K,L). The rationale was that two smaller balloons would induce less invagination of the overhanging stent segment while generating a greater telescoping effect, as both could partially advance into the vessel. However, the technique is complex, difficult to reproduce in vivo, and requires a 7 Fr guiding catheter, multiple devices, and forceful push‐pull maneuvers. Although it produced the most effective longitudinal crush observed to date, optical coherence tomography (OCT) imaging revealed a substantial metallic burden at the ostium, with the telescoped segment remaining markedly malapposed (Figure 2). Additional postdilatation to correct this malapposition led to re‐elongation of the stent and negated much of the achieved foreshortening.

### 5.3 Guidewire Crossing Through a Side Cell of the Stent

Following loss of guidewire access, late recognition of ostial stent protrusion, or when intentional stent correction is planned, re‐crossing through a side cell is often considerably easier than through the true stent lumen, as discussed above. This configuration offers a potentially favorable strategy: the creation of a new functional lumen through the stent's side cells while leaving the protruded segment in place but displaced laterally. The following section examines this approach, focusing on the mechanical behavior of a newly created lateral neo‐lumen, including its maximum achievable diameter and its potential effects on overall stent integrity. Importantly, modern stent platforms demonstrate side‐cell expansion capacities comparable to those of the main stent lumen, allowing safe dilation through the side cell in carefully selected cases [21]. It is important to consider stent design, as some platforms incorporate extra proximal connectors to resist longitudinal compression, which markedly limits side‐cell expansion and may complicate ostial post‐dilatation. Therefore, side‐cell recrossing should be reserved for cases of pronounced stent protrusion and performed exclusively through the most distal cell at the aorto‐ostial interface.

### 5.4 The Side Flap Technique

**FIGURE 3:**
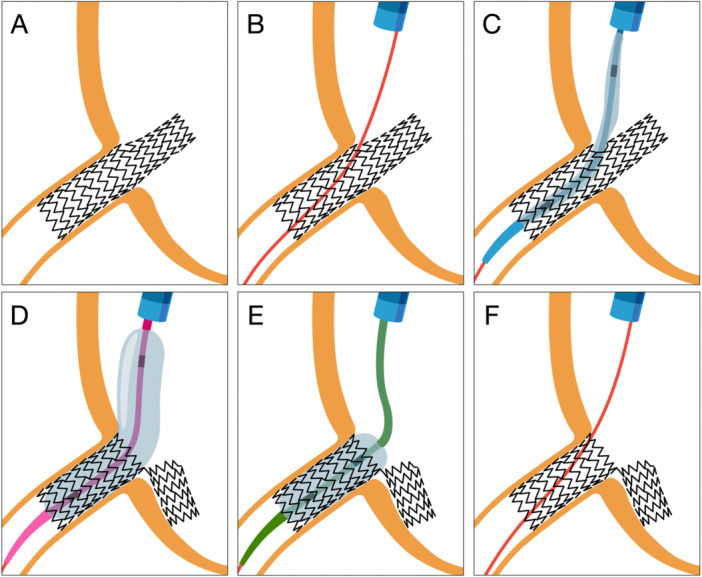
Illustration of the Side Flap Technique (Central Illustration). Illustration of the individual steps of the Side Flap technique. (A) Accidental implantation of a stent with excessive aorto‐ostial stent protrusion, (B) rewiring through a side cell near the true ostium, (C) advancement of a semi‐compliant (SC) balloon through the side cell, (D) predilatation of the side cell using an SC balloon at nominal size and pressure, (E) postdilatation with an non‐compliant (NC) balloon 0.5 mm larger than the ostial diameter at high pressure, (F) final result after postdilatation, showing the protruding segment displaced laterally, forming the characteristic Side Flap configuration. [Color figure can be viewed at wileyonlinelibrary.com]

**FIGURE 4:**
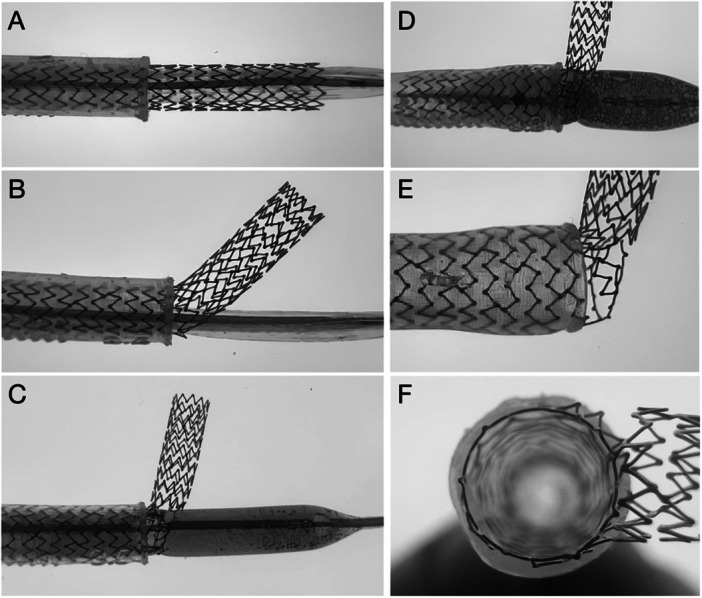
Bench demonstration of the Side Flap technique. Bench demonstration of the individual steps of the Side Flap technique. (A) Implantation of a Synergy Megatron 4.0/32 mm stent with excessive aorto‐ostial protrusion, (B) rewiring through a side cell near the true ostium and passage of a small balloon, (C) predilatation of the side cell with a 4.0 mm SC balloon, (D) postdilatation with a 5.5 mm NC balloon at high pressure, (E) final result after postdilatation showing the protruded segment displaced laterally (Side Flap configuration), (F) ostial view demonstarting excellent stent expansion and strut apposition.

The mirrored provisional approach intentionally creates a new central lumen through a side cell of the protruding stent. In this configuration, the protruded segment is displaced laterally, opposite to the site of side cell crossing (Figure 3, Central illustration). The key determinant of procedural success is the maximum expansion capacity of the side cell. Most contemporary DES platforms feature only two to three connectors per ring; thus, balloon inflation through a single side cell generates a large neo‐lumen without exerting significant traction or torsional force on adjacent segments. This results in a stable and well‐defined neo‐ostium, free of distortion. Balloon expansion from within the coronary lumen further protects the stent portion facing the vessel wall and prevents unwanted deformation (Figure 4).

Although concern may arise regarding the presence of a stent segment protruding into the aortic sinus, experience from transcatheter aortic valve implantation (TAVI) suggests this configuration is well tolerated. Neither sinus sequestration by the valve frame nor the chimney technique, where long stent segments extend into the aorta, has been associated with adverse outcomes [25, 26].

**FIGURE 5:**
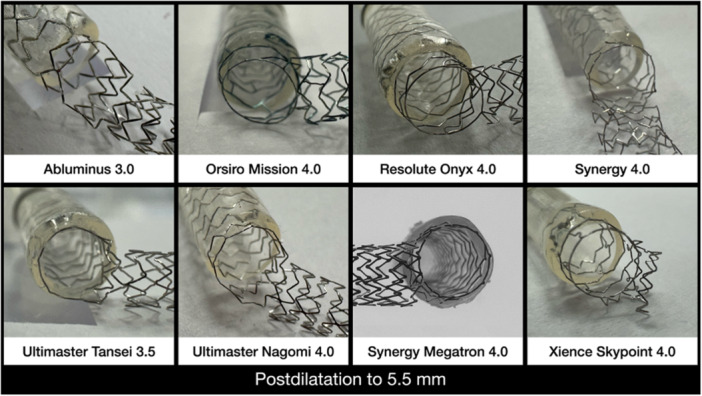
Bench test results of contemporary stent platforms using the Side Flap technique. Bench evaluation of the Abluminus, Orsiro Mission, Resolute Onyx, Synergy, Ultimaster Tansei, Ultimaster Nagomi, Synergy Megatron, and Xience Skypoint stent platforms after application of the Side Flap technique. All stents (3.0‐3.5 mm) were post‐dilated with a 5.5 mm non‐compliant (NC) balloon at 12 atm to assess the maximal expansion capacity of the side cell. Across all platforms, a well‐defined circular neo‐lumen was achieved, with the distal 1‐2 rings maintaining structural integrity and no evidence of strut fracture. The displaced flap was consistently oriented opposite to the re‐wiring site, and visual inspection confirmed complete ring expansion and optimal strut apposition. [Color figure can be viewed at wileyonlinelibrary.com]

The Side Flap technique was tested on all major contemporary DES platforms (Figure 5). Stents with diameters of 3.0 mm and 3.5 mm were evaluated, and all neo‐lumens were post‐dilated with a 5.5 mm NC balloon inflated to 12 atm to determine the maximum side‐strut expansion capacity. Remarkably, all DES tolerated this high‐pressure dilation without structural failure [27, 28]. The resulting neo‐lumens were consistently circular and well defined, with the most proximal one to two rings (depending on the re‐crossing site) maintaining uniform geometry and showing no evidence of strut fracture. In all cases, the displaced flap was directed opposite to the rewiring site.

**FIGURE 6:**
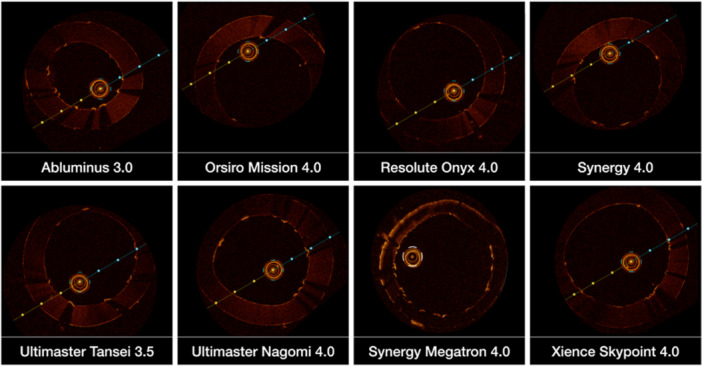
Assessment of ostial stent apposition after the Side Flap technique. Optical coherence tomography (OCT) images demonstrating complete and symmetrical expansion of the newly created neo‐lumen following the Side Flap technique. Cross‐sectional and longitudinal views (not shown) demonstrate optimal strut apposition at the coronary ostium with no evidence of malapposition, strut fracture, or longitudinal deformation. [Color figure can be viewed at wileyonlinelibrary.com]

Optical coherence tomography (OCT) imaging confirmed full circular stent expansion with excellent apposition and no evidence of deformation (Figure 6). These findings support the structural integrity and potential long‐term durability of the Side Flap technique, while also suggesting improved accessibility for future catheter engagement. To minimize residual ostial protrusion after Side Flap creation, distal re‐crossing at the aorto‐coronary ostium is of particular importance.

### 5.5 Snare‐and‐Rupture Technique

**FIGURE 7:**
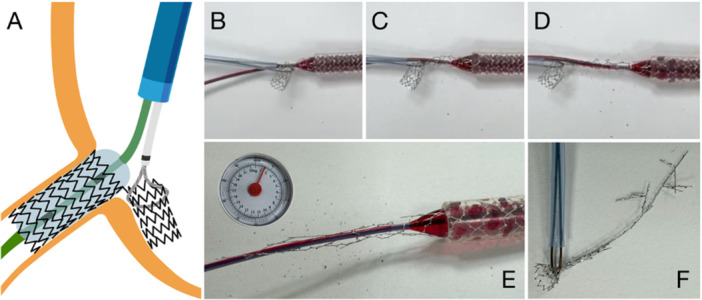
Illustration and Bench images of the Snare‐and‐Rupture technique. (A) Fixation of the stent within the coronary artery using a balloon. (B) Positioning of a goose‐neck snare around the protruding stent segment. (C) Traction applied to withdraw the protruding portion from the ostium. (D). Progressive stent elongation under traction. (E) Pulling forces exceeding ~1.5 kg leading to stent rupture. (F) Detached elongated stent fragment; the rupture point is unpredictable, and residual stent elements may remain at the ostium. [Color figure can be viewed at wileyonlinelibrary.com]

To eliminate the laterally displaced flap, a balloon can be inflated to anchor the stent at the ostium, while the protruding segment is snared and ruptured via the same or a second guiding catheter (ping‐pong technique). The inflated balloon provides internal stabilization of the stent and protection of the vessel wall at the ostium, allowing selective removal of the overhanging portion (Figure 7).

**FIGURE 8:**
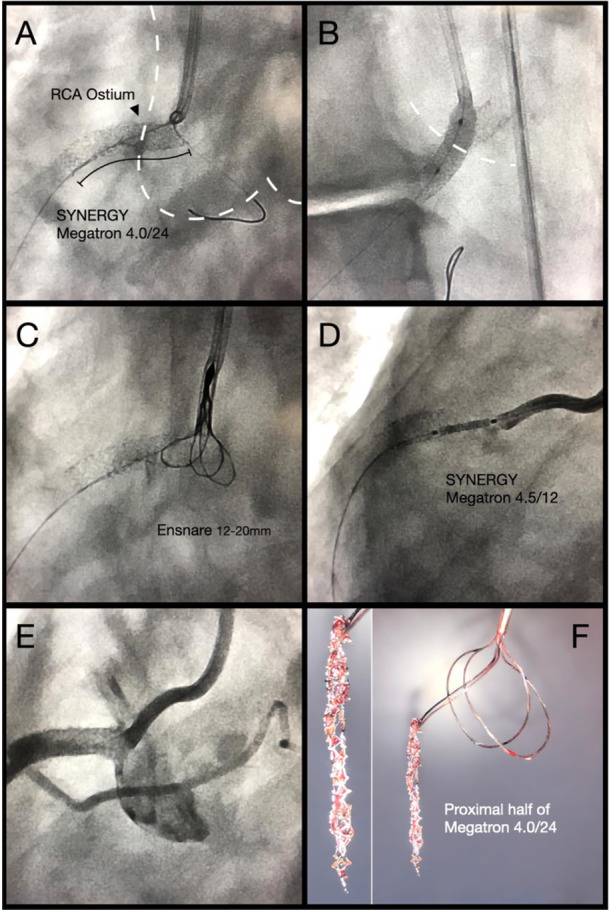
Clinical case of the Snare‐and‐Rupture technique. (A) Side cell crossing. (B) Side flap. (C) Snaring the protruding portion with an Ensure snare. (D) Placement of an additional stent at the RCA ostium. (E) final angiography reveals Side Flap and flush ostial stenting. (F) explanted portion of the protruding stent. [Color figure can be viewed at wileyonlinelibrary.com]

However, the procedure remains highly unpredictable and carries a considerable risk of vessel injury, including dissection and perforation. Bench testing demonstrated that stent rupture required considerable traction forces, reaching up to 1.5 kg depending on the design and type of DES. Furthermore, the anchoring balloon does not adequately secure the implanted portion, leading to pronounced elongation of the entire structure during extraction attempts. Stent rupture may occur in an uncontrolled manner, potentially causing elongation or migration of metallic fragments into the aortic lumen, where visualization is limited, and embolization of polymer coating or metallic debris. It is conceivable, however, that in vivo, particularly in the presence of heavy calcification at the ostium, stent retention could be greater than under bench conditions. Figure 8 illustrates a clinical case in which this technique was applied safely. Nevertheless, given its inherent unpredictability and procedural risks, this method should not be recommended for routine clinical use.

### 5.6 Complete Stent Explantation

**FIGURE 9:**
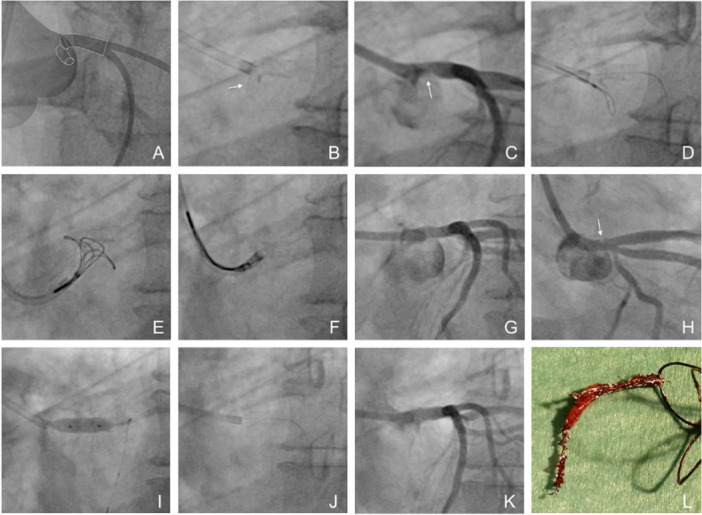
Clinical case of complete stent explantation. Illustrative example of the complete removal of a recently implanted ostial stent. The procedure involves anchoring and traction of the stent en bloc, resulting in full extraction of the metallic scaffold. Despite inevitable elongation, the entire stent can typically be retrieved safely, allowing for subsequent optimal ostial re‐stenting. This technique is feasible only in freshly implanted or short stents before endothelialization has occurred and carries potential risks of vessel dissection, perforation, or aortic injury. [Color figure can be viewed at wileyonlinelibrary.com]

If the stent has been freshly or recently implanted, complete explantation may be attempted [29]. This approach is most suitable for short stents deployed during the same procedure or within a few days, before endothelialization occurs. In such cases, complete removal is preferable to partial extraction, as even when elongation occurs, the entire stent can typically be retrieved safely, permitting optimal subsequent ostial PCI [30]. While complete explantation offers a more definitive solution than partial removal in carefully selected cases, the maneuver remains unpredictable and carries substantial procedural risks, including vessel dissection, perforation, aortic injury, or stent embolization. The operator should be prepared with a second guidewire to promptly re‐cross the vessel following stent removal. Figure 9 illustrates a clinical example where a protruding stent was removed from the ostial LM a few days after implantation.

## 6 Clinical Experience With the Side Flap Technique

In a retrospective multicenter analysis, we identified 15 cases in which the Side Flap technique was applied. Procedural characteristics and clinical follow‐up of all in vivo cases where the Side Flap technique was used are summarized in Table 1. In eight cases (53.3%), excessive protrusion impeded or prevented guide engagement. In one of these, the protruding stent was side‐flapped and subsequently extracted with a goose‐neck snare after balloon fixation in the ostial RCA. Another case involved protrusion at the ostium of a saphenous vein graft rather than a native coronary artery. In two cases (13.3%), the protruding stent interfered with distal percutaneous intervention. In three cases (20%), the protrusion caused ischemia due to marked intimal hyperplasia or thrombus in the protruding part. In one case (6.7%), side‐flapping was required to facilitate transcatheter aortic valve replacement (TAVR) in a patient with a low RCA takeoff. Finally, in one case (6.7%), the Side Flap technique was necessary after retrograde crossing of an RCA CTO through a side cell with subsequent antegrade conversion. The Side Flap procedures were performed 8.8 ± 9.0 months after initial stent implantation. Ostial stenting was necessary in 8 (53.3%) of the cases. Clinical follow‐up was 35.8 ± 31.4 months, with a single patient having a clinically driven TVR due to an in‐stent restenosis (ISR) in another stent in the distal RCA. Of note, none of the patients had any events related to the Side Flap. These observations indicate that the technique achieves favorable procedural success and sustained clinical outcomes in real‐world practice.

**TABLE 1:** Technical and clinical outcomes of the Side Flap technique.

Case	Vessel involved	Size of index DES implanted (mm)	Side Flap balloon diametr (mm)	Reason for Side Flap	Time from index PCI to Side Flap (month)	Additional ostial stent implanted	Follow‐up (month)	Events (TLF, stroke, MI, deah)
1	LM	Xience Skypoint 3.5	4.5	Excessive ostial protrusion	30	Yes	42	None
2	RCA	Xience Skypoint 4.0	4.0	Ostial TLF	24	Yes	28	None
3	RCA	Resolute Onyx 3.0	4.0	Ostial TLF	15	Yes	31	None
4	LM	Orsiro mission 4.0	5.0	Excessive ostial protrusion	6	No	19	None
5	RCA	Resolute Onyx 4.0	5.0	Excessive ostial protrusion	1	Yes	6	None
6	LM	Synergy XD 4.5	4.5	Excessive ostial protrusion	3	No	7	None
7	LM	Synergy Megatron 3.5	6.0	Excessive ostial protrusion	3	No	19	None
8	RCA	Synergy Megatron 4.0	4.5	Snare‐and‐rupture technique	3	Yes	57	None
9	RCA	Xience Sierra 4.0	4.5	Ostial TLF	4	Yes	56	TVR due to ISR distal in another stent
10	RCA	Resolute Integrity 4.0	4.5	Side cell crossing after retrograde CTO‐PCI	4	Yes	44	None
11	RCA	Synergy Megatron 4.0	4.5	Excessive ostial protrusion	4	Yes	36	None
12	RCA	Resolute Onyx 3.0	5.0	complicated distal PCI	5	No	18	None
13	RCA	Xience Skypoint 4.0	4.5	TAVI with low RCA ostium	3 days	No	6	None
14	LM	Resolute Onyx 3.5	5.0	complicated distal PCI	12	No	36	None
15	SVG	Resolute Integrity 3.0	4.0	Excessive ostial protrusion	18	No	132	None

**FIGURE 10:**
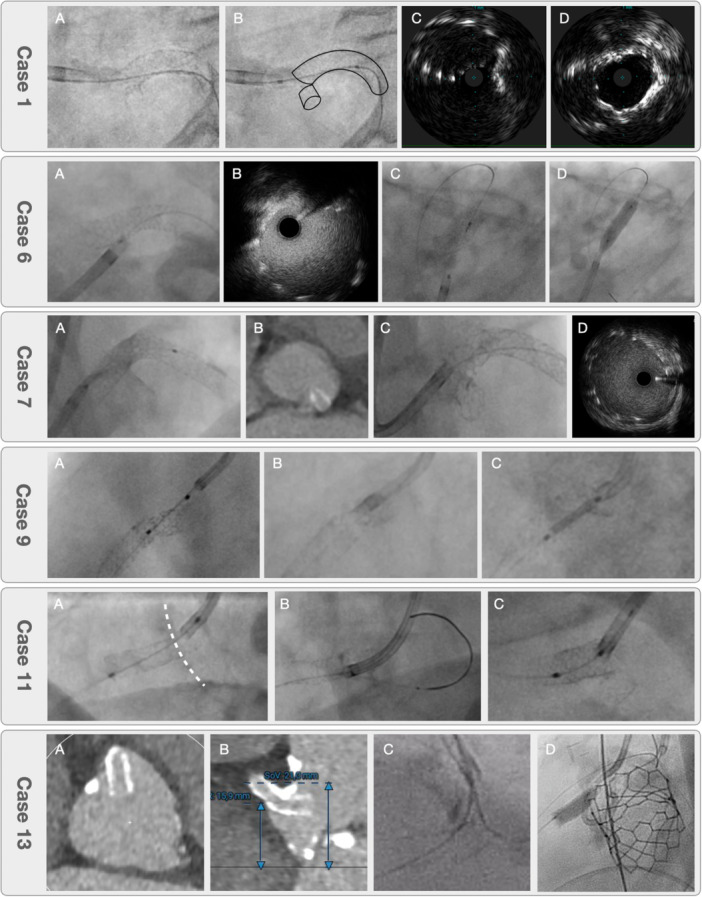
Clinical cases of the Side Flap technique. Representative examples of excessive aorto‐ostial stent protrusion treated with the Side Flap technique. Case 1: (A) cranial rewiring with Side Flap toward aortic valve, (B) black line illustrates the stent and Side Flap, (C) IVUS documentation of ostial side cell rewiring, (D) final IVUS proofs ostial stent apposition. Case 6: (A) dashed line indicates true RCA ostium, (B) WALPO indicates true ostium after side cell rewiring, (C) dashed circle indicates Side Flap. Case 7: (A) dashed circle indicates Side Flap at left main ostium, (B) IVUS confirms ostial stent coverage and apposition, (C) crossing of the side cell with the initial small balloon, (D) POT of the ostium. Case 9: (A) excessive stent protrusion at the left main ostium, (B) Stent protrusion detected by non‐ECG‐triggered CT scan, (C) Side Flap, (D) IVUS confirmation of ostial stent expansion and apposition. Case 11: (A) creation of the Side Flap with a large balloon after ostial side cell crossing, (B) advancement of the guide to the true RCA ostium, (C) placement of an additional stent after Side Flap with partial uncoverage of the RCA ostium. Case 13: (A) cross sectional view in the CT scan shows extensive ostial stent protrusion, (B) axial planes shows the low height of the RCA ostium and high‐risk of stent crush by the calcified right coronary cusp during TAVR, (C). illustrates a stabilizing WALPO wire and the interventional guidewire crossing a distal side cell, (D) Following the initial Side Flap and successful valve implantation, ostial post‐dilatation was performed to optimize stent expansion and exclude any obstruction from the Side Flap. [Color figure can be viewed at wileyonlinelibrary.com]

Figure 10 shows representative procedural images of protruding stents, where the Side Flap technique was applied. Pre‐ and post‐interventional images demonstrate the conversion of the protruding stent into a laterally displaced flap, with restoration of a central neo‐lumen and preservation of flow. In one patient, stent protrusion was incidentally detected on a non‐ECG‐triggered CT scan performed for non‐cardiac reasons, prompting referral for ostial correction. The clinical case series also includes three cases in which IVUS guidance was used to optimize ostial rewiring and confirm accurate positioning before Side Flap creation.

In one patient, protrusion was incidentally detected on a non‐ECG‐triggered CT scan performed for non‐cardiac reasons. In approximately 50% of the cases, an additional stent was placed at the ostium.

## 7 Discussion

There is currently no expert consensus on the optimal management of excessive aorto‐ostial stent protrusion. Reported cases have typically been handled using anecdotal or improvised methods, none of which have been tested systematically. This study sought to address this gap by conducting structured bench experiments for each proposed technique to elucidate the mechanical behavior of both interventional equipment and protruded stents under realistic procedural conditions.

The main findings of our study were: (1) Achieving an optimal longitudinal stent compression is technically challenging, if not impossible. (2) The Side Flap technique proved to be the most controlled, reproducible, and mechanically stable method. (3) The Side Flap technique demonstrated no evidence of periprocedural complications and no long‐term hazard in our clinical series.

In addition, our results also argue against anecdotal approaches aimed at longitudinal stent compression using either the guiding catheter or a large balloon, as these fail to transmit force coaxially and instead produce severe stent deformation and malapposition. The findings also highlight the unpredictability and procedural risks associated with partial stent explantation.

Ostial stent deformation is far more frequent than generally appreciated and arises from two main mechanisms: (1) Immediate deformation during the index PCI, typically due to underexpansion within a calcified or suboptimally prepared lesion, or inadvertent contact with the guiding catheter or other devices; and (2) Acquired deformation due to repetitive hinge motion or fatigue‐related stent fracture, particularly in the right coronary artery [31]. When combined with stent protrusion, such deformation makes accurate re‐wiring challenging, and operators may inadvertently advance the guidewire through a side cell rather than the true stent lumen [29].

Our in vivo experience suggests that the Side Flap technique is an effective option for managing excessive stent protrusion. This approach may be considered in cases where protrusion exceeds 5 mm or when fluoroscopy reveals non‐coaxial guiding catheter engagement, indicative of significant stent overhang. Implantation of an additional short ostial stent can be reserved for selected cases of dissection or insufficient ostial coverage, although bench testing indicates that this is often not required.

Alternative strategies, such as intentional stent extraction, remain occasionally practiced [30] but present substantial challenges in real‐world settings. Extraction leaves the vessel unprotected by a guidewire and often results in immediate flow compromise due to intimal injury [32]. Once the stent is snared, the entire system must be withdrawn en bloc, as the snare becomes entangled with the stent struts [33]. In many cases, the stent cannot be fully retracted into the guiding catheter and becomes lodged at the sheath entry site, requiring sheath removal or a small skin incision, resulting in complete loss of vascular access [34]. Hwang et al. demonstrated that stent design significantly affects extraction behavior: three‐connector stents tend to elongate and are more likely to be retrieved intact, whereas two‐connector designs are prone to fracture during traction [35].

While the Side Flap technique has proven safe and mechanically stable, it is not entirely devoid of risk. In cases of very large protrusion, the displaced segment may contact the aortic valve, and rare reports exist of cusp perforation due to repeated contact between the stent and the valve [36]. In such scenarios, the Side Flap technique may further displace the stent toward the valve, warranting meticulous procedural planning. Side‐wiring should be performed with particular care, ideally from a lateral or even inferior direction, to minimize this risk. Damarkusuma et al. were among the first to describe an unintentional application of the Side Flap technique in a case involving a 9.1 mm protruded RCA stent [37]. The operators realized they had crossed through a side cell only after balloon dilation and discontinued the procedure due to concern for stent fracture and embolization. The patient developed restenosis 3 months later, at which time a second stent was successfully implanted through the newly created neo‐lumen. Remarkably, the patient remained free of thrombosis and maintained vessel patency for 10 years before requiring target lesion revascularization (TLR).

In cases of short stent protrusion ( < 5 mm), a brief and deliberate attempt at true‐lumen wiring, preferably using a dual‐lumen microcatheter, should remain the initial strategy. The Side Flap technique can be reserved for excessive stent protrusions in which true‐lumen access cannot be achieved or is not technically feasible, while recognizing that side‐cell expansion may be limited in chronically embedded or calcified stents.

Our bench studies have several limitations. The mechanical behavior of a DES evaluated in vitro may differ from that observed in vivo, where stent struts are frequently embedded within neo‐intimal tissue, atherosclerotic plaque, or calcified segments. In such settings, side‐cell expansion may be constrained by the incorporation of the stent ring into surrounding tissue, limiting strut mobility and occasionally requiring rotational atherectomy to ablate the obstructing strut. Incomplete circular expansion of the neo‐lumen is more commonly observed with first‐generation DES, due to unfavorable side‐cell expansion. Any degree of stent deformation increases the risk of stent thrombosis and recurrent ISR. In light of these considerations, a brief and deliberate attempt at rewiring the central true stent lumen, without stent crushing, remains an appropriate initial strategy to restore luminal patency.

Although excessive stent protrusion itself may contribute to aortic valve injury, chronic repetitive trauma to the aortic valve cusps caused by the flapped, previously protruding stent segment could not be evaluated in this experimental model and therefore represents a potential limitation of the Side Flap technique.

## 8 Conclusion

The Side Flap technique represents a practical and a reproducible alternative for the management of excessive aorto‐ostial stent protrusion. Its use should be considered in light of inherent procedural limitations and the need for further clinical validation.

## Conflicts of Interest

**Gregor Leibundgut:** consulting/speaker/proctoring honoraria from Ashai Intecc, Biotronik, Teleflex, Orbus Neich, Terumo, Novartis. **M. Bilal Iqbal:** funding, honoraria and consulting fees from Astrazeneca, Novartis, St. Judes, Teleflex, Boston Scientific, Shockwave Medical, Biotronik, Terumo Medical, Asahi Intecc, APT Medical **Claudiu Ungureanu:** no conflicts of interest to declare. **Anastasios Barmpas:** no conflicts of interest to declare. **Jasper Boeddinghaus:** is supported by an Edinburgh Doctoral College Scholarship and received research grants from the University of Basel, the University Hospital of Basel, the Division of Internal Medicine, the Swiss Academy of Medical Sciences, the Gottfried and Julia Bangerter‐Rhyner Foundation, the Swiss National Science Foundation, the Swiss Heart Foundation, and honoraria from Siemens, Roche Diagnostics, Ortho Clinical Diagnostics, Quidel Corporation, and Beckman Coulter, and travel support from Medtronic and Vascularmedical, all outside the submitted work. **Teodor Buliga:** no conflicts of interest to declare. **Gabor G Toth:** consulting/speaker/proctoring honoraria from Biotronik, Medtronic, Boston Scientific, Abbott and Terumo. **Alexandru Achim:** no conflicts of interest to declare.
